# The Dark Triad and the PID-5 Maladaptive Personality Traits: Accuracy, Confidence and Response Bias in Judgments of Veracity

**DOI:** 10.3389/fpsyg.2017.01549

**Published:** 2017-09-21

**Authors:** Benno G. Wissing, Marc-André Reinhard

**Affiliations:** ^1^Department of Psychology, Cognitive Psychology, University of Kassel Kassel, Germany; ^2^Department of Psychology, Social Psychology, University of Kassel Kassel, Germany

**Keywords:** dark triad, PID-5, detachment, deception, confidence judgments, response bias

## Abstract

The Dark Triad traits—narcissism, Machiavellianism and psychopathy—have been found to be associated with intra- or interpersonal deception production frequency. This cross-sectional study (*N* = 207) investigated if the Dark Triad traits are also associated with deception detection accuracy, as implicated by the recent conception of a deception-general ability. To investigate associations between maladaptive personality space and deception, the PID-5 maladaptive personality traits were included to investigate if besides Machiavellianism, Detachment is negatively associated with response bias. Finally, associations between the Dark Triad traits, Antagonism, Negative Affectivity and confidence judgments were investigated. Participants watched videos of lying vs. truth-telling senders and judged the truthfulness of the statements. None of the Dark Triad traits was found to be associated with the ability to detect deception. Detachment was negatively associated with response bias. Psychopathy was associated with global confidence judgments. The results provide additional support that dark and maladaptive personality traits are associated with judgmental biases but not with accuracy in deception detection. The internal consistencies of 4 of the 8 subscales of the used personality short scales were only low and nearly sufficient (αs =0.65–0.69).

## Introduction

Research on the Dark Triad (Paulhus and Williams, 2002)—the moderately intercorrelated personality traits of narcissism, Machiavellianism and psychopathy—has accumulated over the recent years. Within the two dominant personality frameworks, the Five-Factor Model (FFM; Costa and McCrea, 1992) and the HEXACO model (Lee and Ashton, 2005), they converge on a core of low agreeableness (Paulhus and Williams, 2002) and low Honesty-Humility (Lee and Ashton, 2005). On the interpersonal level, individuals high in Dark Triad traits are more agentic and lower in communion (Jonason et al., 2010; Jones and Paulhus, 2010) reflecting a low manifestation of agreeableness, which is defined as the willingness to cooperate and, therefore, of central importance for group inclusion (Buss, 1991).

Narcissism is characterized by grandiosity, entitlement, dominance and superiority (Raskin and Hall, 1979; Corry et al., 2008); Machiavellianism is associated with a cold, cynical, amoral worldview and detached, strategic manipulativeness (Christie and Geis, 1970); psychopathy is linked with impulsivity, risk-taking, low neuroticism and low empathy (Hare, 1985). Some authors argue for the domain specific adaptiveness of the Dark Triad traits (e.g., Jonason et al., 2014a).

The existence of different fitness consequences for different personality traits in different environmental niches, suggests that “dark” or “maladaptive” personality traits may represent frequency-dependent fitness optima in certain environmental niches (Penke et al., 2007). Fitness and social desirability are distinct concepts (Nettle, 2006). For example, high Antagonism might be adaptive in exploitable and exploitative environments, exemplified by individuals high in Machiavellianism who disregard conventional morality by rationally defecting when defection is the equilibrium strategy (Gunnthorsdottir et al., 2002).

The integration of individual differences in terms of deception ability within an evolutionary framework varies with its definition of deception ability as a target space for natural selection, as either divisible into deception production and detection ability (Mealey, 1995) or indivisible, conceptualized as a deception-general ability (Wright G. R. T. et al., 2012). In the former case, the dyadic dynamics of a co-evolutionary arms race between predatory, defecting cheaters and cooperators arises (Dawkins and Krebs, 1979; Mealey, 1995)—cheater detection is conceptualized as an evolved mechanism to protect against exploitation in social exchange situations (Cosmides and Tooby, 1992)—, in the latter case “wizards” of deception detection *and* production should result. Empirical support for a deception-general ability currently only exists in the form of found negative correlations between detectability as sender and discrimination ability as receiver (*r*s = −0.35, −0.47; Wright G. R. T. et al., 2012; Wright et al., 2015).

Overall, the data suggest, that humans are only slightly better than chance, at detecting deception (Bond and DePaulo, 2006) and truth-biased in their response, i.e., they believe in the truthfulness of others independently of their actual truthfulness (Levine et al., 1999). On the level of judging deception, humans differ more in response bias than in actual ability (Bond and DePaulo, 2008). Data on the relationship between personality and deception detection accuracy is sparse (Aamodt and Custer, 2006). This sparsity seems to be particularly present concerning the study of dark personality traits and is additionally amplified in terms of the interpretation of results by studies investigating a singular trait without controlling for the shared variance of dark personality traits, e.g., the Dark Triad traits. The results in the existing literature are mixed. For instance, in men, primary psychopathy has been found to be correlated with lie detection ability (Lyons et al., 2013), other studies did not find an association (e.g., Peace and Sinclair, 2012). Also, no superior lie detection ability for Machiavellianism has been found (Zuckerman et al., 1981), but in woman Machiavellianism has been found to be associated with lie detection ability (Lyons et al., 2017). A recent study found no association between Dark Triad traits and deception detection or deception production ability in an interactive deception task (Wright et al., 2015). While the ecological validity of the study (Wright et al., 2015) might be high in comparison with classical studies of deception detection based on audiovisual stimulus material, the statistical power is questionable given the relatively small sample size (*N* = 75) and necessitates further investigation.

Recent research on the Dark Triad traits and active deception indicates that individuals high in Dark Triad traits, particularly those scoring high on the more antagonistic traits of Machiavellianism and psychopathy, differ more from individuals low in Dark Triad traits in deception production frequency (higher for Machiavellianism and psychopathy; Kashy and DePaulo, 1996: Baughman et al., 2014; Jonason et al., 2014b) and amplitude (high-stakes deception for Machiavellianism; Azizli et al., 2016). In contrast, narcissism is primarily associated with self-deception (e.g., Paulhus and Williams, 2002), theorized as an evolutionary evolved intrapersonal mechanism to assist interpersonal deception (von Hippel and Trivers, 2011). The data on the relation between the Dark Triad traits and self-reported lying skills are mixed (Baughman et al., 2014; Jonason et al., 2014b), but suggest overall that the Dark Triad traits are associated with self-perceived deceptive abilities (Giammarco et al., 2013).

Based on findings in the cognitive branches of psychology and neuroscience, the proposed deception-general ability is centered around the associations of executive functions and theory of mind—the ability to understand others' mental states—with deception production and deception detection ability (Wright G. R. T. et al., 2012). Among the Dark Triad traits, grandiose narcissism is exclusively positively associated with theory of mind (Vonk et al., 2015), whereas Machiavellianism and psychopathy are both associated with deficits in empathy and theory of mind (Ali et al., 2009; Ali and Chamorro-Premuzic, 2010; Vonk et al., 2015). Narcissism has also been found to predict self-estimated mind-reading performance (Ames and Kammrath, 2004).

Linked with dark personality traits are maladaptive ones (Grigoras and Wille, 2017). Section III of the fifth edition of the *Diagnostic and Statistical Manual of Mental Disorders* (DSM-5; American Psychiatric Association, 2013) contains an empirically derived, dimensional model of five maladaptive personality traits (PID-5; Krueger et al., 2012) that constitute the maladaptive versions of the normative Five-Factor Model (FFM; Costa and McCrea, 1992) in the following hierarchical order: Negative Affectivity (i.e., emotional lability, anxiousness, separation insecurity; FFM Neuroticism), Detachment (i.e., withdrawal, anhedonia, intimacy avoidance; low FFM Extraversion), Antagonism (i.e., Manipulativeness, Deceitfulness, Grandiosity; low FFM Agreeableness), Disinhibition (i.e., irresponsibility, impulsivity, distractibility; low FFM Conscientiousness), and Psychoticism (i.e., unusual beliefs and experiences, eccentricity, perceptual dysregulation; FFM Openness; Thomas et al., 2012). Links of the PID-5 traits with narcissism (Wright et al., 2013) and psychopathy (Strickland et al., 2013; Anderson et al., 2014) are established and highlight Antagonism as the central factor. The PID-5 traits have been shown to outperform the Big Five as predictors of the Dark Triad traits (Grigoras and Wille, 2017).

One possible solution to the often-unsuccessful linkage of personality and deception may be to specifically capture the meta-analytically distilled differences on multiple levels (e.g., modality, sender) on the level of receiver-personality. For the most significant individual difference—sender credibility (Bond and DePaulo, 2008)—this has been realized on the level of the receiver by suspiciousness, which has been shown to decrease truth bias if experimentally induced (e.g., McCornack and Levine, 1990; Millar and Millar, 1997; Kim and Levine, 2011). Suspiciousness is one facet of PID-5 Detachment, but the Detachment domain could contain deception relevant features beyond suspiciousness. Meta-analytically extracted modality-based differences in deception detection accuracy and response bias (Bond and DePaulo, 2006) can be interpreted to some extent as the result of modality-mediated differences in detachment between sender and receiver (Burgoon et al., 2008). These differences in sender-receiver-detachment may not be entirely modality-based, but may be partially determined by Detachment on the level of receiver-personality. Therefore, the PID-5 domain of Detachment could play an important role on the level of personality for the process of deception detection.

In this study, the relations between the Dark Triad traits, the PID-5 maladaptive personality traits, and the process of lie detection including detection accuracy, response bias and confidence judgments and process measures for self-reported cue reliance and self-reported decision time were investigated.

Based on the associations of Machiavellianism with a cynical worldview (Christie and Geis, 1970; Jones and Paulhus, 2009), negative views of others (Mudrack, 1993; Rauthmann and Will, 2011; Rauthmann, 2012) and lie acceptability (Wright et al., 2015), Machiavellianism was predicted to be negatively associated with response bias.

Based on its relation with suspiciousness and its potential sender-receiver-detachment enhancing function, Detachment was predicted to be negatively associated with response bias.

Based on the association of the Dark Triad traits with intra- or interpersonal deception production frequency and based on the assumption that frequency is associated with ability, given that deception production ability can be trained (Verschuere et al., 2011; Hu et al., 2012) and, finally, based on the association of deception production ability and deception detection ability (Wright G. R. T. et al., 2012; Wright et al., 2015) it was predicted that the Dark Triad traits are associated with deception detection accuracy.

Based on the negative association of the Dark Triad traits with agreeableness and humility and previous findings regarding self-perceived deceptive competence (Giammarco et al., 2013), it was predicted that the Dark Triad traits are linked to local and global confidence judgments.

Based on the grandiose aspects of Antagonism, it was predicted that Antagonism is associated with local and global confidence judgments. Based on the negative association of Neuroticism with confidence (e.g., Burns et al., 2016), it was predicted that Negative Affectivity is negatively associated with local and global confidence judgments.

## Materials and methods

The study was conducted in full accordance with the Ethical Guidelines of the German Association of Psychologists (DGPs) and the American Psychological Association (APA). Moreover, by the time the data were acquired it was also not required at Kassel University, nor at most other German universities to seek ethics approval for simple studies on personality and attitudes. The study exclusively makes use of anonymous questionnaires. No identifying information was obtained from participants. The participants were explicitly informed that the data are treated confidentially. Every participant had to agree to the following statements: “I understand that my participation is voluntary and that I may withdraw from the study at any time without explanation;” and “I hereby confirm that I am at least 18 years old, and that I agree to take part in this study.” Furthermore, they could withdraw from the study at any time.

### Statistical power and participants

Based on effects sizes from previous studies for deception detection accuracy (Bond and DePaulo, 2006) and response bias (Bond and DePaulo, 2008), we estimated a lower sample size bound of *N* = 176/204 based on a small to medium effect size of *f*2 = 0.1 with *k* = 3/5 predictors, α = 0.05, Power 1–β = 0.95 using the statistical power analysis tool G^*^Power (Faul et al., 2009). Given only the small meta-analytically identified interindividual differences in accuracy (Bond and DePaulo, 2006), this effect size may still be appointed too high for accuracy, but considering the proposed general-deception ability (Wright G. R. T. et al., 2012) it seems more reasonable in the context of traits associated with high intra- or interpersonal deception frequency—given the assumption that frequency is associated with ability, given that deception production ability can be trained (Verschuere et al., 2011; Hu et al., 2012)—and the estimated sample size is a significant improvement in terms of statistical power over the original study (*N* = 75; Wright et al., 2015).

Participants that dropped out before finishing the deception detection task were excluded from data analysis, resulting in the final sample of 207 participants (59.9% female; *M* age = 29.03; *SD* age = 10.62, age range = 17–66) that were recruited from Germany using online invocations and invitations on the campus of the university of Kassel. The study was conducted online and lasted for a total of approximately 25 min.

### Procedure and measures

Personality was assessed using the German version of the *Naughty Nine* short scale, a 9-item psychometrically optimized version of the *Dirty Dozen* (Jonason and Webster, 2010) self-report instrument, which measures the Dark Triad traits with good internal consistency and stability (Küfner et al., 2015), consisting of narcissism (e.g., “*I tend to want others to admire me*”; α = 0.82), Machiavellianism (e.g., “*I tend to manipulate others to get my way*”; α = 0.75) and psychopathy (e.g., “*I tend to lack remorse*”; α = 0.69) using a 9-point assumed interval-type scale (1 = *disagree strongly*, 9 = *agree strongly*), followed by the German version of the *Personality Inventory for DSM-5 Brief Form* (*PID-5-BF*; Krueger et al., 2012; American Psychiatric Association, 2013), that measures the five maladaptive dimensional personality trait domains of the PID-5 model with 25 items, consisting of Negative Affectivity (e.g., “*I worry about almost everything*”; α = 0.68), Detachment (e.g., “*I often feel like nothing I do really matters*”; α = 0.65), Antagonism (e.g., “*It's no big deal if I hurt other peoples' feelings*”; α = 0.72), Disinhibition (e.g., “*People would describe me as reckless*”; α = 0.69), and Psychoticism (e.g., “*My thoughts often don't make sense to others*”; α = 0.77) using a 4-point Likert-type scale (0 = *very false/often very false*, 3 = *very true/often true*). The US and Danish version of the PID-5-BF have shown acceptable internal consistencies (Anderson et al., 2016; Bach et al., 2016). The full 220-item version of the PID-5 is currently validated in German (Zimmermann et al., 2014).

To measure deception detection accuracy, participants were randomly assigned to one of two video sets. Each video set consisted of 14 videos, in one half of which the sender was telling the truth, whereas telling lies in the other half, so that the information that was depicted (honestly vs. dishonestly) was balanced across the videos assigned to different participants. 4 out of 14 senders of each video set were female. The videos displayed an employment interview situation, where the candidate (sender) was asked a question by the interviewer (receiver), whereas only the candidate was visible and the camera perspective simulated the point of view of the interviewer. The entire body of the sender was visible and the interviewer was blind to the experimental conditions. Each sender was instructed to convince the interviewer of a job they had in the past vs. one they had not worked in in the past. Every sender was only visible in one of the two video sets (further information on the audiovisual stimulus material can be found in Reinhard et al., 2013). To measure deception detection accuracy, the participants were instructed to decide, whether the candidate was telling the truth or lying after watching each video. After each binary truth vs. lie decision was made, the participants were asked how confident they were in their judgment (e.g., “*How confident are you in your judgment?*”; α = 0.84) using a continuous percental-type scale (0% = *absolutely uncertain*, 100% = *absolutely certain*). This process was repeated for the totality of all 14 videos. Truth bias was used as the response bias measure and was determined by the total number of truth judgments.

After completion of the deception detection task, self-reported decision time was measured (e.g., “*When did you decide on the truthfulness of the candidate?*”) by using an 11-point assumed interval-type scale (0 = *directly at the beginning of the video*, 10 = *after completion of the video*). Thereafter, self-reported verbal and nonverbal cue reliance were measured on a 10-point assumed interval-type scale (1 = *strongly disagree*, 10 = *strongly agree*) with two items each (e.g., “*I focused on the content*,” “*I used the content of the statements for my judgment*”; α = 0.94; “*I focused on the nonverbal behavior*, “*I used the nonverbal behavior for my judgment*”; α = 0.90). Finally, to measure global confidence, the participants were asked to estimate their overall detection accuracy in absolute terms (e.g., “*How many of the 14 videos do you think you judged correctly?*”; 0–14).

## Results

Because of multiple comparisons, a significance threshold of α = 0.01 was used to reduce Type I errors. All statistical statements are relating to the data, not the theory. Data analysis was conducted with R (R Core Team, 2017).

### Prespecified data analysis

#### Response bias

Zero-order correlations and standardized regression weights for the personality variables and response bias can be seen in Tables 1, 2. The participants were truth-biased [*M* = 64.63%, *SD* = 17.45; *M*Δ = 14.63, 95% *CI* = (12.24, 17.02); *t*_(206)_ = 12.06, *p* < 0.001]. The data did not support the predicted negative association of Machiavellianism with response bias [*r*_(205)_ = −0.05, 95% *CI* = (−0.19, 0.08), *p* = 0.448]; However, the predicted negative association between Detachment and response bias was supported by the data [*r*_(205)_ = −0.23, 95% *CI* = (−0.36, −0.10), *p* < 0.001].

**Table 1:** Zero-order correlations and standardized regression weights with 95% CIs (in brackets) for the Dark Triad and deception variables.

	***R^2^* (*f^2^*)**	**Narcissism**	**Machiavellianism**	**Psychopathy**	**Dark Triad composite**
**DECEPTION DETECTION ACCURACY**					
Overall	0.00 (−0.01; 0.00; 0.02)	−0.03 (−0.19; −0.03; 0.13)	−0.02 (−0.16; 0.01; 0.17)	−0.05 (−0.19; −0.05; 0.10)	−0.05
Truth	0.02 (−0.02; 0.02; 0.07)	0.00 (−0.13; 0.03; 0.18)	−0.06 (−0.18; −0.02; 0.14)	−0.15 (−0.30; −0.15; −0.01)	−0.09
Lie	0.01 (−0.02; 0.01; 0.05)	−0.03 (−0.22; −0.06; 0.10)	0.03 (−0.13; 0.03; 0.19)	0.11 (−0.04; 0.11; 0.25)	0.05
Response bias	0.03 (−0.02; 0.03; 0.07)	0.02 (−0.11; 0.05; 0.21)	−0.05 (−0.19; −0.03; 0.13)	−0.15 (−0.30; −0.15; −0.01)	−0.08
**CONFIDENCE JUDGMENTS**					
Local	0.02 (−0.02; 0.03; 0.07)	0.06 (−0.14; 0.02; 0.17)	0.11 (−0.09; 0.07; 0.23)	0.14 (−0.03; 0.11; 0.25)	0.14
Global	0.05 (0.00; 0.06; 0.13)	0.12 (−0.10; 0.05; 0.21)	0.16 (−0.08; 0.09; 0.25)	0.20^*^ (0.03; 0.17; 0.31)	0.22^*^
Over	0.05 (−0.01; 0.05; 0.12)	0.12 (−0.09; 0.06; 0.21)	0.15 (−0.09; 0.07; 0.23)	0.20^*^ (0.03; 0.17; 0.31)	0.21^*^

N = 207;

* *p < 0.01 (two-tailed)*.

**Table 2:** Zero-order correlations and standardized regression weights with 95% CIs (in brackets) for the PID-5 traits and deception variables.

	***R^2^* (*f^2^*)**	**Negative Affectivity**	**Detachment**	**Antagonism**	**Disinhibition**	**Psychoticism**
**DECEPTION DETECTION ACCURACY**						
Overall	0.01 (−0.02; 0.01; 0.05)	−0.05 (−0.22; −0.06; 0.10)	−0.02 (−0.15; 0.01; 0.17)	−0.09 (−0.25; −0.10; 0.05)	0.01 (−0.10; 0.05; 0.21)	−0.04 (−0.18; −0.01; 0.17)
Truth	0.06 (0.00; 0.07; 0.14)	0.04 (0.00; 0.16; 0.31)	−0.21^*^ (−0.35; −0.19; −0.04)	−0.08 (−0.16; −0.01; 0.13)	−0.07 (−0.19; −0.04; 0.11)	−0.13 (−0.27; −0.10; 0.08)
Lie	0.08^*^ (0.01; 0.09; 0.18)	−0.10 (−0.38; −0.23^*^; −0.07)	0.19^*^ (0.06; 0.21^*^; 0.37)	−0.02 (−0.24; −0.10; 0.05)	0.09 (−0.05; 0.10; 0.24)	0.09 (−0.08; 0.09; 0.26)
Response bias	0.09^*^ (0.02; 0.10; 0.20)	0.08 (0.07; 0.23^*^; 0.38)	−0.23^**^ (−0.39; −0.24^*^; −0.09)	−0.04 (−0.09; 0.05; 0.19)	−0.10 (−0.23; −0.08; 0.07)	−0.13 (−0.28; −0.11; 0.06)
**CONFIDENCE JUDGMENTS**						
local	0.03 (−0.01; 0.03; 0.08)	−0.14 (−0.29; −0.13; 0.03)	−0.07 (−0.21; −0.05; 0.10)	0.07 (−0.05; 0.10; 0.24)	−0.04 (−0.15; 0.00; 0.15)	−0.06 (−0.18; 0.00; 0.17)
global	0.05 (0.00; 0.06; 0.13)	−0.12 (−0.22; −0.07; 0.09)	−0.12 (−0.26; −0.10; 0.05)	0.12 (0.03; 0.17; 0.32)	−0.02 (−0.12; 0.04; 0.19)	−0.13 (−0.28; −0.11; 0.06)
over	0.05 (−0.01; 0.05; 0.12)	−0.08 (−0.18; −0.02; 0.13)	−0.09 (−0.25; −0.09; 0.06)	0.15 (0.06; 0.20^*^; 0.35)	−0.02 (−0.15; 0.00; 0.15)	−0.09 (−0.26; −0.09; 0.08)

Note: N = 207;

* p < 0.01

** *p < 0.001 (two-tailed)*.

**Figure 1:**
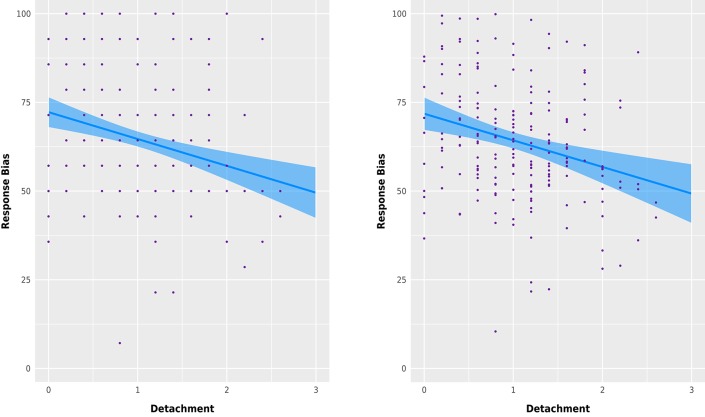
Robust linear regression with 95% CI for response bias as a function of Detachment with *k* = 1 predictor **(left)** and all *k* = 5 dimensions of PID-5 maladaptive personality space as predictors **(right)**.

A regression model with Detachment as the predictor variable and response bias as criterion variable, suggested substantial heteroscedasticity of the residuals. Therefore, a robust regression with MM-estimator was computed. As seen in Figure 1, Detachment emerged as a substantial predictor [*b* = −7.57, 95% *CI* = (−11.42, −3.71)] that predicted a response bias range of ŷ = [49.54, 72.25]; With all PID-5 traits entered as predictor variables into the model, Detachment emerged as the only substantial predictor [*b* = −7.50, 95% *CI* = (−11.97, −3.04)] and showed no substantial difference in its association pattern with the criterion variable.

Linear model assumptions were validated including by using the R package gvlma (Pena and Slate, 2014). The robust regression model was computed by using the R packages MASS (Venables and Ripley, 2002), robust (Wang et al., 2017), and prediction (Leeper, 2017). Figure 1 was constructed with the R package visreg (Breheny and Burchett, 2017) and the ggplot2 (Wickham, 2009) plotting engine.

#### Deception detection accuracy

Overall detection accuracy was *M* = 50.38% (*SD* = 11.00) and not different from chance [*t*_(206)_ = 0.50, *p* = 0.620]. Zero-order correlations and standardized regression weights for the personality variables with measures of deception detection accuracy can be seen in Tables 1, 2. The data did not support the predicted association between the Dark Triad traits and deception detection accuracy.

#### Confidence judgments

Zero-order correlations and standardized regression weights for the personality variables with measures of confidences judgments can be seen in Tables 1, 2. Among the Dark Triad traits, a substantial association with confidence judgments emerged only for psychopathy and global confidence judgments [*r*_(205)_ = 0.20, 95% *CI* = (0.07, 0.33), *p* = 0.004]. Antagonism and Neuroticism were not substantially associated with confidence judgments.

### Exploratory data analysis

#### Personality

Intercorrelations of the personality traits can be seen in Table 3. As expected based on prior research, all individual Dark Triad traits were associated most strongly with Antagonism. Overall, the correlational pattern is in line with the one found in a previous study with longer measures of the Dark Triad traits and the PID-5 traits (Grigoras and Wille, 2017). Personality scales were computed with the R package psych (Revelle, 2017).

**Table 3:** Descriptive statistics and zero-order correlations for the Dark Triad traits and the PID-5 traits.

	**α**	***M* (*SD*)**	**1**	**2**	**3**	**4**	**5**	**6**	**7**	**8**	**9**
**DARK TRIAD**											
1. Narcissism	0.82	4.83 (1.92)	–								
2. Machiavellianism	0.75	3.97 (1.90)	0.48^**^	–							
3. Psychopathy	0.69	3.17 (1.77)	0.13	0.32^**^	–						
4. Dark Triad composite	0.78	3.99 (1.37)	0.75^**^	0.82^**^	0.64^**^	–					
**PID-5**											
5. Negative Affectivity	0.68	1.28 (0.59)	0.15	0.09	−0.21^*^	0.02	–				
6. Detachment	0.65	1.04 (0.61)	−0.09	0.10	0.22^*^	0.10	0.30^**^	–			
7. Antagonism	0.72	0.60 (0.50)	0.35^**^	0.62^**^	0.54^**^	0.68^**^	0.08	0.25^**^	–		
8. Disinhibition	0.69	0.83 (0.55)	0.00	0.14	0.16	0.13	0.35^**^	0.27^**^	0.21^*^	–	
9. Psychoticism	0.77	1.02 (0.67)	0.11	0.26^**^	0.06	0.20^*^	0.47^**^	0.46^**^	0.28^**^	0.38^**^	–

N = 207;

* p < 0.01

** *p < 0.001 (two-tailed)*.

#### Truth and lie detection accuracy

Zero-order correlations and standardized regression weights for the personality variables and truth and lie detection accuracy can be seen in Tables 1, 2. Accuracy for truth detection was above chance [*M* = 65.01%, *SD* = 21.05; *M*Δ = 15.01, 95% *CI* = (12.13, 17.89); *t*_(206)_ = 10.26, *p* < 0.001]. Detachment was negatively associated with truth detection accuracy [*r*_(205)_ = −0.21, 95% *CI* = (−0.33, −0.07), *p* = 0.003]. When controlling for response bias in a first-order partial correlation, the confidence interval for the associations between Detachment and truth detection accuracy included zero [*r*_(204)_ = −0.01, 95% *CI* = (−0.15, 0.12), *p* = 0.850]. Accuracy for lie detection was below chance [*M* = 35.75%, *SD* = 20.20; *M*Δ = −14.25, 95% *CI* = (−17.02, −11.48); *t*_(206)_ = −10.15, *p* < 0.001]. Detachment was positively associated with lie detection accuracy [*r*_(205)_ = 0.19, 95% *CI* = (0.05, 0.32), *p* = 0.006]. When controlling for response bias in a first-order partial correlation, the confidence interval for the associations between Detachment and lie detection accuracy included zero [*r*_(204)_ = −0.01, 95% *CI* = (−0.15, 0.12), *p* = 0.850]. First-order partial correlations were computed with the R package psych (Revelle, 2017).

#### Confidence judgments

To further explore confidence judgments and deception detection performance, a measure of overconfidence was computed by subtracting global confidence judgments (the number of self-estimated correct judgments) by the number of actual accurate judgments. As can be seen in Tables 1, 2, psychopathy and the Dark Triad composite were associated with overconfidence. Antagonism emerged as a predictor of overconfidence.

#### Self-reported process measures

Personality variables were not substantially associated with self-reported process measures. In line with previous findings (Reinhard et al., 2011), self-reported verbal cue reliance was associated with overall deception detection accuracy [*r*_(205)_ = 0.22, 95% *CI* = (0.08, 0.34), *p* = 0.002].

## Discussion

The present study investigated the relation between the Dark Triad traits, the PID-5 maladaptive personality traits and the process of lie detection including detection accuracy, response bias, confidence judgments and process measures for self-reported cue reliance and self-reported decision time.

There was no association of Dark Triad traits with the ability of deception detection in the data. This finding is in line with previous research (e.g., Wright et al., 2015), that found no relation of the Dark Triad traits with deception detection ability. Instead, and also in line with previous findings (Giammarco et al., 2013), an association of psychopathy and the Dark Triad composite with global confidence judgments appeared in the data. More importantly, the global confidence judgments were not grounded in actual deception detection performance: On average, individuals with higher psychopathy and overall Dark Triad scores reported higher confidence in their deception detection accuracy than their actual accuracy permitted—they were overconfident in their ability. The confidence interval of the standardized regression coefficients for psychopathy suggested, that psychopathy alone could account for unique variance in global confidence and overconfidence above the shared variance of the Dark Triad. This pattern is in line with findings of a recent meta-analysis, that psychopathy is often the only significant correlate of important psychosocial outcomes, if the shared variance of the Dark Triad is controlled (Muris et al., 2017). The absence of a substantial association pattern between Machiavellianism and response bias should be interpreted cautiously, given the relatively high prior probability in the form of the well-established connection between Machiavellianism and interpersonal suspiciousness.

On the level of maladaptive personality, Detachment emerged as a predictor of response bias with a minus-signed coefficient. Effect sizes for response bias predicted by Detachment can be considered substantial in the context of the criterion construct, given that humans are generally truth-biased and that very high Detachment scores predicted the absence of response bias. In a meta-analysis with 32 samples, a mean observed standard range of 50.06% in response bias was found (Bond and DePaulo, 2008). The response bias range predicted by Detachment was 22.71%, which corresponds to 45.37% of the meta-analytically mean observed standard range in response bias.

While the truth-default mode is likely to be adaptive in environments, where most communication is honest (Levine, 2014), Detachment may facilitate adaptive behavior in environments with high deception frequency by providing a lower or even no response bias, and therefore, a higher lie detection accuracy. In such environments, the interpersonally active core of PID-5 Detachment—withdrawal from other people—may not be maladaptive, but serve a protective function. This potential adaptive function in environmental niches with high deception frequency is contrasted by the finding that within PID-5 maladaptive personality space, facets of Detachment and Negative Affectivity exhibit the strongest connections with a general index of personality disorder severity (Hopwood et al., 2012).

### Limitations and future research

The present study was based on short self-report measures. The self-reported measures of cue reliance and decision time are inherently subjective. It is highly questionable, if the subjects had cognitive access to their cue reliance modalities, but self-reported cue reliance is associated with objective outcomes, e.g., verbal cue reliance is associated with deception detection accuracy (Reinhard et al., 2011). On the level of personality assessment, the short Naughty Nine instrument revealed an anomalistic intercorrelation pattern of the Dark Triad traits, in which Machiavellianism was more strongly associated with narcissism than with psychopathy. The PID-5-BF instrument can only measure maladaptive personality on the level of domains and can potentially produce a higher measurement error in subclinical samples (Krueger and Markon, 2014). Psychopathy, Negative Affectivity, Detachment, and Disinhibition had questionable internal consistencies (αs = 0.65–0.69). The internal consistency coefficients are in line with the corresponding validation studies, e.g., αs = 0.57–0.76 for psychopathy (Küfner et al., 2015) and the validation study of the US version of the PID-5-BF found two alpha coefficients below α = 0.70 (Anderson et al., 2016). Overall, the intended optimization of the trade-off between reliability and the prevention of fatigue effects in the process of deception detection was not sufficiently successful. Beyond these limitations, the generalizability of the findings to real-world contexts of deception detection is questionable. Furthermore, the non-significant results could represent type II errors resulting from power deficiency, given that a true effect exits with a smaller size than estimated. Future studies should therefore aim to use longer instruments with higher internal consistencies and larger samples to increase statistical power.

In the current PID-5 model, suspiciousness is a facet of both Detachment and Negative Affectivity, but the replicated finding of marginal secondary loadings of suspiciousness on Negative Affectivity questions the relationship of the facet with its higher-order factor (Wright A. G. C. et al., 2012; De Fruyt et al., 2013; Zimmermann et al., 2014). An interesting question is, if facets of Detachment beyond suspiciousness can account for variance in response bias. Future research should use longer versions of the PID-5, which can measure the facets of Detachment. Beyond the facet level resolution, the interactions between modality-based and personality-based differences in Detachment are worth investigating. Are there levels of Detachment on the level of personality that can account for modality-based differences in sender-receiver-detachment for response bias and deception detection accuracy? In situations where cues are impairing deception detection accuracy, individuals high in Detachment may find it easier to ignore these unreliable cues to deception.

The relation of the Dark Triad traits and deception ability could be investigated in experimental settings, that provide a more optimal fit regarding Dark Triad specific motivations and affordances, e.g., given that situational familiarity enhances deception detection accuracy (Reinhard et al., 2011). The Dark Triad traits should express their antagonistic behaviors specifically in selfishness vs. cooperation scenarios (Rauthmann, 2012), driven by the shared psychogenic motivational core of power (Kajonius et al., 2015; Jonason and Ferrell, 2016). Future studies should, therefore, strive to activate the power motive by providing incentives for power acquisition via deception production or deception detection in contexts, that provide selfish vs. cooperative behavioral optionality.

## Statement for disclosure of sample, conditions, measures, and exclusions

The authors confirm that they have reported all measures, conditions, data exclusions, and how they determined their sample size.

## Code, data and materials

Code, data and materials can be accessed via: https://osf.io/tcy3q/.

## Author contributions

All authors listed have made a substantial, direct and intellectual contribution to the work, and approved it for publication.

### Conflict of interest statement

The authors declare that the research was conducted in the absence of any commercial or financial relationships that could be construed as a potential conflict of interest.
